# Draft Genome Sequence of the First NDM-1-Producing *Providencia stuartii* Strain Isolated in Portugal

**DOI:** 10.1128/genomeA.01077-15

**Published:** 2015-09-24

**Authors:** Vera Manageiro, Daniel A. Sampaio, Patrícia Pereira, Paulo Rodrigues, Luís Vieira, Carlos Palos, Manuela Caniça

**Affiliations:** a National Reference Laboratory of Antibiotic Resistances and Healthcare Associated Infections, Department of Infectious Diseases, National Institute of Health Dr. Ricardo Jorge, Lisbon, Portugal; b Centre for the Studies of Animal Science, Institute of Agrarian and Agri-Food Sciences and Technologies, Oporto University, Oporto, Portugal; c Innovation and Technology Unit, Department of Human Genetics, National Institute of Health, Dr. Ricardo Jorge, Lisbon, Portugal; d Hospital Beatriz Ângelo, Loures, Portugal

## Abstract

We report here the draft genome sequence of the first NDM-1-producing *Providencia stuartii* strain isolated in Portugal. Sequence analyses revealed the presence of an incompatibility group A/C2 (IncA/C2) plasmid and of diverse acquired genes conferring resistance to β-lactams, aminoglycosides, tetracycline, macrolides, chloramphenicol, and sulfonamides. This sequence contributes to the evaluation of the spread of NDM-1 producers.

## GENOME ANNOUNCEMENT

*Providencia stuartii*, an opportunistic pathogen typically associated with urinary infections, has intrinsic resistance to antibiotics considered to be of last resort, such as colistin and tigecycline (1, 2). Moreover, carbapenemase producers have been reported, which can become of higher concern (3–5).

Five NDM-1-producing *P. stuartii* isolates were obtained during an outbreak detected in a Portuguese hospital. One isolate, *P. stuartii* INSRA21868, recovered from a urine sample from an 88-year-old male patient admitted to the intensive care unit due to an enterocutaneous fistula, was selected for genetic characterization using whole-genome sequencing (WGS).

DNA was extracted from an overnight grown culture using the QIAamp DNA minikit (Qiagen), according to the manufacturer’s instructions. Libraries were prepared from 1 ng of genomic DNA using the Nextera XT DNA sample preparation kit (Illumina), according to the manufacturer’s instructions. WGS was performed using 150-bp paired-end reads on a MiSeq (Illumina). The sequence reads were trimmed and filtered according to quality criteria and *de novo* assembled into contigs and subsequently into scaffolds by means of CLC Genomics Workbench 8.0.1 (Qiagen, Aarhus, Denmark).

The analysis yielded 59 contigs ranging from 246 bp to 365,814 bp, with a minimum of 100-fold coverage. The draft genome contains a total assembly length of 4,438,644 bp, with a mean coverage of about 290-fold; the G+C content was 43.1%.

All *de novo* contigs were searched against the GenBank database nucleotide collection (nr/nt) using Mega BLAST, with seven contigs (15/41/43/47/48/49/53) mapping against plasmid sequences deposited there; the remaining sequence aligned to the *P. stuartii* genome (accession no. NZ_CP008920 and CP003488).

The nucleotide sequence was annotated using the NCBI Prokaryotic Genome Annotation Pipeline (PGAP) (http://www.ncbi.nlm.nih.gov/genome/annotation_prok/). The numbers of annotated features were as follows: 3,962 coding sequences (CDSs), 4,146 genes, 20 noncoding RNAs (ncRNAs), 4 rRNAs, and 72 tRNAs.

*In silico* antimicrobial resistance analyses using ResFinder version 2.1 (6), with a threshold of 90% identity and a minimum length of 40%, revealed genes conferring resistance to β-lactams (*bla*_NDM-1_ [contig 53], *bla*_CMY-4_ [contig 15], and Δ*bla*_DHA-1_ [contig 53]), aminoglycosides (*aac(2′)-Ia* [contig 19] and *armA* [contig 49]), tetracycline (*tetB* [contig 9]), macrolides (*mphE* and *msrE* [contig 49]), chloramphenicol (*catB3* [contig 2]), and sulfonamides (*sul1* [contig 49]). PlasmidFinder version 1.2 (7) analyses revealed the presence of incompatibility group A/C2 (IncA/C2), which has been associated with a wide dissemination of *bla*_NDM-1_.

The NDM-1-encoding gene was found in the 3,360-bp-long contig 53, with a mean coverage of about 136-fold and a G+C content of 57.9%. In the 3′ region, the *bla*_NDM-1_ gene was adjacent to a bleomycin resistance-encoding gene (*ble*_MBL_), followed by a *trpF* and part of the *bla*_DHA-1_-*ampR* region. The IS*Aba125* element upstream of *bla*_NDM-1_ was interrupted by an IS*26* element. Four plasmids harbored by different *Enterobacteriaceae* isolates showed the highest identities with this genetic structure (accession numbers KJ187752, LN831185, JX988621, and HQ451074).

Overall, this genome sequence contributes to the evaluation of the global molecular epidemiology and spread of NDM-1 producers.

### Nucleotide sequence accession numbers.

This whole-genome sequencing shotgun project has been deposited at DDBJ/EMBL/GenBank under the accession no. LGYB00000000. The version described in this paper is version LGYB01000000.
